# Evaluation of health-related quality of life in hemolytic uraemic syndrome patients treated with eculizumab: a systematic evaluation on basis of EMPRO

**DOI:** 10.1080/0886022X.2018.1427110

**Published:** 2018-01-24

**Authors:** Anwesha A. Mukherjee, Amit D. Kandhare, Subhash L. Bodhankar

**Affiliations:** Department of Pharmacology, Poona College of Pharmacy, Pune, India

**Keywords:** Eculizumab, evaluating measures of patient Reported outcomes, hemolytic uraemic syndrome, health-related quality of life, patient-reported outcomes, systematic review

## Abstract

**Background:** Hemolytic uraemic syndrome (HUS) is progressive renal failure disease and determination of their quality of life (QoL) on the basis of patient-reported outcomes (PROs) are becoming increasingly important in the economic evaluations for its treatment with eculizumab (ECU).

**Aim:** To perform the systematic evaluation of QoL in HUS patients treated with ECU on the basis of Evaluating Measures of Patient Reported Outcomes (EMPRO) tool.

**Materials and methods:** A systematic review was conducted in PubMed, EMBASE, the Cochrane Library, CINAHL and Google Scholar till September 2016 by two independent researchers. Each identified instrument was evaluated for its quality of performance by using the EMPRO tool for its overall score and seven attribute specific scores (range 0–100, worst to best).

**Results:** Five different PROs instruments were identified from 10 articles (*n* = 112) which showed eculizumab significantly improves health-related quality of life (HRQOL) in atypical HUS (aHUS) patients. Amongst five instruments viz. EuroQol five dimensions questionnaire (EQ-5 D), Functional Assessment of Chronic Illness Therapy-Fatigue (FACIT-F), Headache Impact Test-6 (HIT-6), 36-Item Short Form Health Survey (SF-36) and Visual Analogue Scale (VAS), the overall EMPRO score was higher for VAS (73.83) and EQ-5 D (73.81). Whereas, FACIT-F and HIT- 6 were just able to meet the minimal threshold of EMPRO scoring (50.24 and 59.09, respectively).

**Conclusions:** Evidence from present investigation support that eculizumab significantly improves HRQoL in patients with aHUS furthermore, EQ-5 D and VAS instrument should be recommended for assessing HRQoL in them. However, selection of PRO instrument for determination of QoL in HUS entirely depend upon the study requirements.

## 1. Introduction

Hemolytic uraemic syndrome (HUS) is progressive renal failure disease clinically characterized by the presence of increased serum urea and creatinine levels, microangiopathic hemolytic anemia and thrombocytopenia [1,2]. Amongst the two different types of HUS, atypical HUS (aHUS) is etiologically distinct and classified as a rare or ultra-orphan disease. The incidence of aHUS is estimated to two per million and prevalence is approximately seven per million in children [3]. A study carried out by Loirat and Frémeaux-Bacchi [4] have shown that aHUS represents 5–10% of HUS in children and is increasingly recognized in adults. Genetic mutations in complement-regulating genes (such as complement factor H, complement factor I and thrombomodulin) has been responsible for aHUS. Despite active treatment, 30% patients develop end-stage renal disease (ESRD) which may lead to death [5,6]. Although the progression of aHUS to ESRD is more frequent in adults (46%) but mortality is higher in children (6.7%) [7,8].

Management of aHUS includes intensive care, dialysis and plasma exchange which helps to decrease mortality in children. However, long-term dialysis options have been very restrictive in patients with ESRD [9,10]. To overcome this issues an initiative has been undertaken for renal transplantation or use of combined liver-kidney transplantation [11,12] however, it is associated with significant morbidity and risk of death [4]. Thus a newer therapy was tested in these patients such as eculizumab which is a recombinant, humanized, a monoclonal IgG2/4κ antibody that targets C5 [11]. In 2011, eculizumab was approved in the US [13] and shortly after that after that in Europe [14]. Now, it is used worldwide for the treatment of aHUS. It is almost uniformly recommended that eculizumab should be started immediately once a patient is diagnosed with aHUS [4].

Quality of life (QoL) plays a vital role in the economic evaluations for any treatment thus its determination is important [15]. Moreover, global QoL of patients is a very useful marker for evaluation of an outcome measure [16,17]. For an array of the disease state, self-reported measures of physical and mental health can be captured by the multidimensional Health-related quality of life (HRQoL) [18–20]. It’s a validated questionnaire that measures various aspects of life such as including physical functioning, psychosocial functioning, role functioning, mental health and general health status [21,22]. The data derived from HRQoL can be used for measuring treatment risk and as well as benefits which may assist in developing interventions to improve patient’s life [23,24].

Nowadays, patient-reported outcomes (PROs) are becoming increasingly important along with the other clinical endpoints and many agencies including the Food and Drug Administration (FDA) recommend the use of PROs for clinical evaluation [25]. Furthermore, PRO measurement needs reliable as well as valid instruments and their selection must be based on the individual study purpose, setting and available resource [26]. Numerous PROs are used in aHUS patients; however, only few are being used consistently [27–29]. To the best of our knowledge, none of the studies have assessed the effect of demographic, clinical, psychological and treatment-related factors on HRQOL in aHUS patients.

Therefore, there is an unmet need for tools capable of capturing all relevant aspects in aHUS patients which is a validated, sensitive and reliable measure to assess disease symptoms, progression and severity. Many attempts have been made to systemize evaluation criteria for PROs. Amongst various tools, two important tools used for PROs evaluation criteria are: the COnsensus-based Standards for the selection of health status Measurement INstruments (COSMIN) [30] and the Evaluating Measures of Patient-Reported Outcomes (EMPRO) [31]. For evaluation of the methodological quality of each study, the COSMIN tool was developed whereas to assess the quality of the PRO, EMPRO tool was developed. EMPRO tool has advantages of obtaining a standardized global score from different instruments and allows to compare between them [31].

EMPRO developers have defined the quality of a PRO measure as the ‘degree of confidence that all possible bias has been minimized and that the information about the process which led to its development and evaluation is clear and accessible’ [31]. EMPRO consist of important aspects including well-depicted and established attributes for assessment, expert reviewers to conduct the assessment and scores that permit a direct comparison of outcome measures. It has been well established that EMPRO is a valid and reliable tool for assessing the performance generic [31] and disease-specific PROs (such as heart failure, shoulder disorders, disease-specific breast cancer and disease-specific prostate cancer) [32–35].

The present study aimed to conduct a systematic review of the effect of eculizumab on HRQoL in aHUS and the standardized EMPRO evaluation of HRQoL instruments that are currently applicable in patients with aHUS disease.

## 2. Materials and methods

This systematic review was conducted in line with the Preferred Reporting Items for Systematic reviews and Meta-Analyses (PRISMA) statement [36,37].

### 2.1. Search strategy

We systematically searched for randomized controlled trials (RCTs) involving articles concerning determinants of HRQOL in aHUS published in English. PubMed (since 1966), EMBASE (since 1966), Cochrane Library (since 1996), PsycInfo (since 1960), CINAHL (since 1982) and Google Scholar were systematically searched for titles and abstracts published between inception date of the database and September 2016. The search strategy contained a combination of keywords (and their synonyms) and Medical Subject Headings (MESH)/EMTREES (in the case of EMBASE), including ‘hemolytic uremic syndrome’, ‘eculizumab’, ‘Quality of Life’ and ‘patient-reported outcome measure’. Spelling variations were also used. Additional articles were obtained through citation snowballing to locate primary sources. We also searched Clinicaltrials.gov to identify ongoing but still unpublished studies.

### 2.2. Study inclusion criteria

Inclusion criteria for the studies of the present investigation were as follows: studies with English language published in a peer-reviewed journal, aHUS patient population which received eculizumab treatment and HRQoL determination with a validated instrument.

Exclusion criteria for this study was as follows: experimental studies, mechanistic studies, commentary, animal studies, letters/reviews/editorials, case series (sample size <10 patients), pharmacodynamic/pharmacokinetic studies, economic evaluation studies and studies with full-text published in a language other than English. Review articles were searched for eligible articles in their reference lists.

Two independent reviewers AM and AK reviewed all retrieved full-text articles depending on inclusion and exclusion criteria. A third arbiter (SB) agreement was obtained in case the eligibility could not be accomplished.

### 2.3. Evaluating Measures of Patient-Reported Outcomes (EMPRO)

The EMPRO [31,35] was designed to measure the quality of PRO instruments. It assesses quality as an overall concept, which is based on eight attributes (39 items) covering: ‘’conceptual and measurement model’ (concepts and population intended to assess); ‘reliability’ (to which degree an instrument is free of random error); ‘validity’ (to which degree an instrument measures what it intends); ‘responsiveness’ (ability to detect change over time); ‘interpretability’ (assignment of meanings to instruments scores); ‘burden’ (time, effort and other demands for administration and response); ‘alternative modes of administration’ (i.e., self- or interviewer-administered, telephone or computer-assisted interview) and ‘cross-cultural and linguistic adaptations’ (equivalence across translated versions). All EMPRO attributes and items are accompanied by a short description to facilitate understanding the intended meaning and to guarantee a standardized application during the evaluation process. The item content of each attribute is summarized in the table of EMPRO results. Agreement with each item can be answered on a four-point Likert’s scale, from 4 (strongly agree) to 1 (strongly disagree). The ‘no information’ box can be checked in case of insufficient information. Five items allow replying with ‘not applicable’. It is recommended to provide detailed comments to justify each EMPRO rating. These comments aid in the better interpretation of the EMPRO scores.

### 2.4. Standardized EMPRO evaluation

Each instrument was evaluated by two appraisers independently and disagreements were resolved either by discussion, or if required, an additional evaluation was performed by a third independent reviewer. Reviewers expressed their degree of agreement on a four-point scale ranging from 4 ‘strongly agree’ to 1 ‘strongly disagree’ for all the items. A further option of ‘no information available’ could be checked if the information in the available document was insufficient to make the decision. For a few items, the option of ‘not applicable’ was also available. Also, reviewers provided detailed comments to explain their ratings. Attribute scores were calculated as the mean of the responses to all items for that attribute, with a linear transformation to obtain the scores on a scale from 0 (minimum) to 100 (maximum).

### 2.5. Quality assessment

Studies were assessed for quality of randomization, blinding, reporting of withdrawals, generation of random numbers and concealment of allocation according to the Cochrane systematic review software (version 5.0.1) [38,39]. This validated Cochrane Risk of Bias tool consisted of the following six categories: (1) random sequence generation; (2) allocation concealment; (3) blinding of participants; (4) incomplete outcome data; (5) selective outcome reporting and (6) other bias. Each category was scored as high, uncertain or low risk of bias. Two independent reviewers (AM, AK) performed the quality assessment and disagreements on scores were resolved through discussion with a third arbiter, SB.

### 2.6. Statistical analysis

Attribute-specific scores and an overall score were calculated. Detailed information and algorithms to obtain EMPRO scores are available online [31,35]. First, the mean of the applicable items was calculated for each attribute (when at least 50% of them were rated) and second, this raw mean was linearly transformed into a range of 0 (worst possible score)–100 (best possible score). Items for which the response option ‘no information’ had been selected were assigned a score of 1 (lowest possible score). Separate sub-scores for the ‘reliability’ and ‘burden’ attributes were calculated as they are composed of two components each: ‘internal consistency and reproducibility’ for reliability, as well as ‘respondent and administrative’ for the burden. For reliability, the highest sub-score for the two components was then chosen to represent the attribute.

Besides the attribute-specific scores, an overall score was computed by calculating the mean of the five metric related attributes: ‘conceptual and measurement model’, ‘reliability’, ‘validity’, ’responsiveness to change’ and ‘interpretability’. The overall score was only calculated when at least three of these five attributes had a score. EMPRO scores were considered reasonably acceptable if they reached at least 50 points (out of the 100 maximum theoretical points). This threshold was chosen based on the global recommendations made by the reviewers in the first two EMPRO studies [31,32]. The receiver operating characteristic (ROC) curve was calculated to evaluate the agreement between EMPRO attribute scores and the reviewers global recommendations.

## 3. Results

### 3.1. Study identification and inclusion

**Figure 1.**
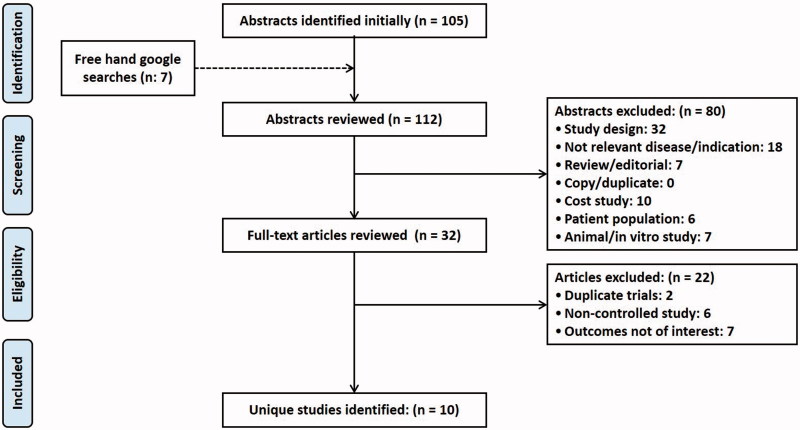
PRISMA flow diagram depicting the selection of studies for systematic review.

Our literature searches initially identified 112 articles after removal of all duplicates. Then they were screened on title and abstract following the predefined inclusion and exclusion criteria. Incorrect study design and a population of interest were the main reasons for article exclusions. In total, 80 articles were excluded, leaving 32 articles which satisfied the inclusion criteria for full-text screening. After full-text review, 10 articles satisfied the inclusion criteria and were included for qualitative analysis [27–29,40–46]. Consensus between the two independent reviewers [AM, AK] was reached in 24% of cases. Figure 1 depicts the PRISMA flow chart of the present study. The primary findings from the included studies are summarized in Table 1. Out of 10 studies, only one study was conducted in the US [40] and remaining were conducted in multiple countries [27–29,41–46].

**Table 1.** Characteristics of included studies (*n* = 10).

Sl. No.	Author, year of publication, country and r number eference	Study characteristics		Characteristics of participants		HRQOL Questionnaire	Results	*p*-value
		Design	Study period (weeks)	Sample size	Mean/median age, years (SD)			
1.	Saultz et al. (2015), USA [40]	P	52	1026	51 (range 19– 69)	HIT- 6	No significant difference in the HIT-6 scores of aHUS patients	–
2.	Muus et al. (2011), Multinational [41]	P, O, S, M, P-2	104	37	28	EQ-5D, VAS	ECU significantly improved the HRQoL in aHUS patients	.002
3.	Licht et al. (2012), Multinational [42]	P, O, S, M, P-2	104	20	28 (range 13–63)	EQ-5D	ECU significantly improved the HRQoL in aHUS patients	<.001
4.	Legendre et al. (2013), Multinational [27]	P, O, S, M, P-2	52	37	28 (range 13–63)	EQ-5D, VAS	ECU significantly improved the HRQoL in aHUS patients	<.001
5.	Licht et al. (2015), Multinational [29]	P, O, S, M, P-2	104	37	28 (range 13–63)	EQ-5D	ECU significantly improved the HRQoL in aHUS patients	<.05
6.	Greenbaum et al. (2014), Multinational [43]	P, O, S, M, P-2	52	22	6.6 (6.1)	FACIT-F	ECU significantly improved the HRQoL in aHUS patients	.0001
7.	Greenbaum et al. (2016), Multinational [28]	P, O, S, M, P-2	26	22	6.5 (0.4–17)	FACIT-F	The 26 weeks of ECU therapy significantly improved the HRQoL in aHUS patients	<.0001
8.	Greenbaum et al. (2012), Multinational [44]	P, O, S, M, P-2	104	17	28 (range 17–68)	EQ-5D	ECU significantly improved the HRQoL in aHUS patients	.0001
9.	Loirat et al. (2011), Multinational [45]	P, O, S, M, P-2	104	17	28 (range 17–68)	EQ-5D	ECU significantly improved the HRQoL in aHUS patients	.0001
10.	Fakhouri et al. (2016), Multinational [46]	P, O, S, M, P-2	26	44	40 (range 18–80)	EQ-5D, FACIT-F and SF-36	ECU significantly improved the HRQoL in aHUS patients	.001

P: prospective; O: open-label; S: single-arm; M: multicenter; P-2: phase 2 trials; EQ-5 D: EuroQol five dimensions questionnaire; FACIT-F: Functional Assessment of Chronic Illness Therapy-Fatigue; SF-36: 36-item Short Form Health Survey; HIT- 6: Headache Impact Test-6; VAS: Visual Analogue Scale; ECU: eculizumab; aHUS: atypical hemolytic-uremic syndrome; HRQoL: Health-Related Quality of Life.

### 3.2. Characteristics of the studies included

Table 1 shows the study characteristics which were included in this study. Totally out of the1279 enrolled patients, only263 (20.56%) patients received eculizumab treatment. Nine studies were open-label and single-arm prospective clinical trial studies [27–29,41–46]. Only one was a prospective clinical trial study [40].

### 3.3. Risk of bias

**Figure 2.**
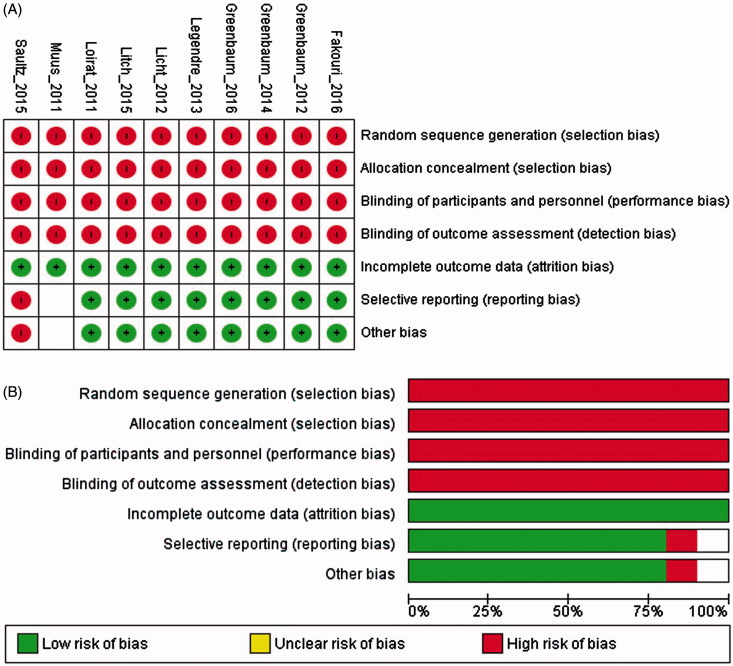
Risk of bias graph of included trials: Review authors’ judgments about each risk of bias item for each included study (A) and each risk of bias item presented as percentages across all included studies (B).

The risk of bias assessment for the included studies are presented in Figure 2. All 10 studies [27–29,41–46] showed a high risk of bias related to random sequence generation, allocation concealment and blinding whereas eight studies [27–29,42–46] showed a low risk of bias for the incomplete collection of outcome data or selective reporting.

Downs and Black scoring were used to evaluate the quality of the studies Using the study quality as a variable. The overall quality of study reporting was good; external validity was better and internal validity was low amongst studies (Table 2). A heat map of an overview of the quality of the studies determined using Downs and the Black scoring system is provided in the Supplementary material.

**Table 2.** Review of the quality of studies determined using downs and black scoring system.

Sl. No.	Author	Reporting (Max: 11)	External validity (Max: 3)	Internal validity-bias (Max: 7)	Internal validity -confounding (selection bias) (Max: 6)	Total (27)
1.	Saultz et al. (2015), USA [40]	5	3	1	0	9
2.	Muus et al. (2011), Multinational [41]	11	3	4	2	20
3.	Licht et al. (2012), Multinational [41]	11	3	4	2	20
4.	Legendre et al. (2013), Multinational [27]	11	3	4	2	20
5.	Licht et al., (2015), Multinational [29]	11	3	4	2	20
6.	Greenbaum et al. (2014), Multinational [43]	11	3	3	1	18
7.	Greenbaum et al. (2016), Multinational [28]	11	3	3	1	18
8.	Greenbaum et al. (2012), Multinational [44]	10	3	3	0	16
9.	Loirat et al. (2011), Multinational [45]	10	3	3	0	16
10.	Fakhouri et al. (2016), Multinational [46]	11	3	4	2	20

### 3.4. Evaluating Measures of Patient-Reported Outcomes (EMPRO)

We identified 32 articles with information concerning five different instruments. After application of the defined exclusion criteria, 22 articles were excluded as they were not instrument related or did not provide any information on the development process, metric properties or administration issues. Finally at the end of the review process., 10 articles which provided information about the PRO measures were included.

**Figure 3.**
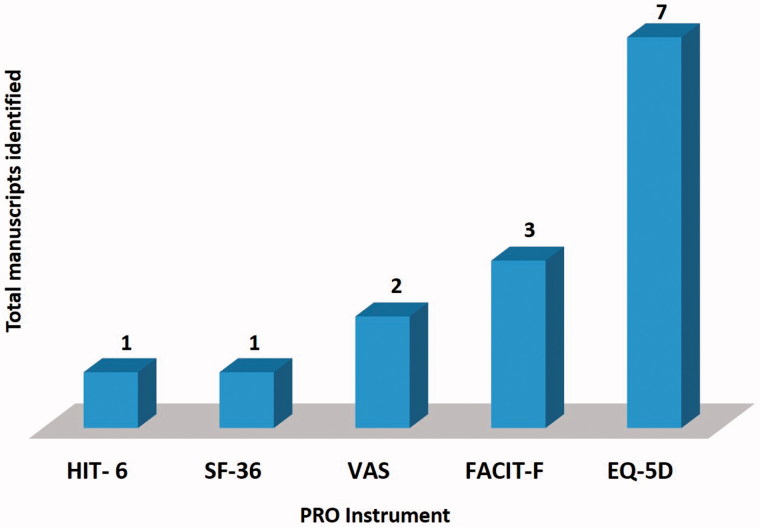
Results of the systematic literature review. Number of manuscripts identified and used in the EMPRO evaluation.

Figure 3 depicts the results of the systematic review process for EMPRO. Seven of the included studies employed generic HRQoL questionnaires i.e., EuroQol five dimensions questionnaire (EQ-5 D) 36-item [27,29,41,42,44–46]. The disease-specific questionnaires, i.e., Functional Assessment of Chronic Illness Therapy-Fatigue (FACIT-F) were utilized in three studies to evaluate the HRQoL [28,43,46]. Another generic unidimensional pain questionnaires, i.e., Visual Analog Scale (VAS) were used in a couple of studies [27,41]. Short-Form Health Survey Questionnaire (SF-36) [46] and Headache Impact Test-6 (HIT-6) [40] were also used to determine HRQoL in aHUS patients.

### 3.5. EMPRO ratings

**Figure 4.**
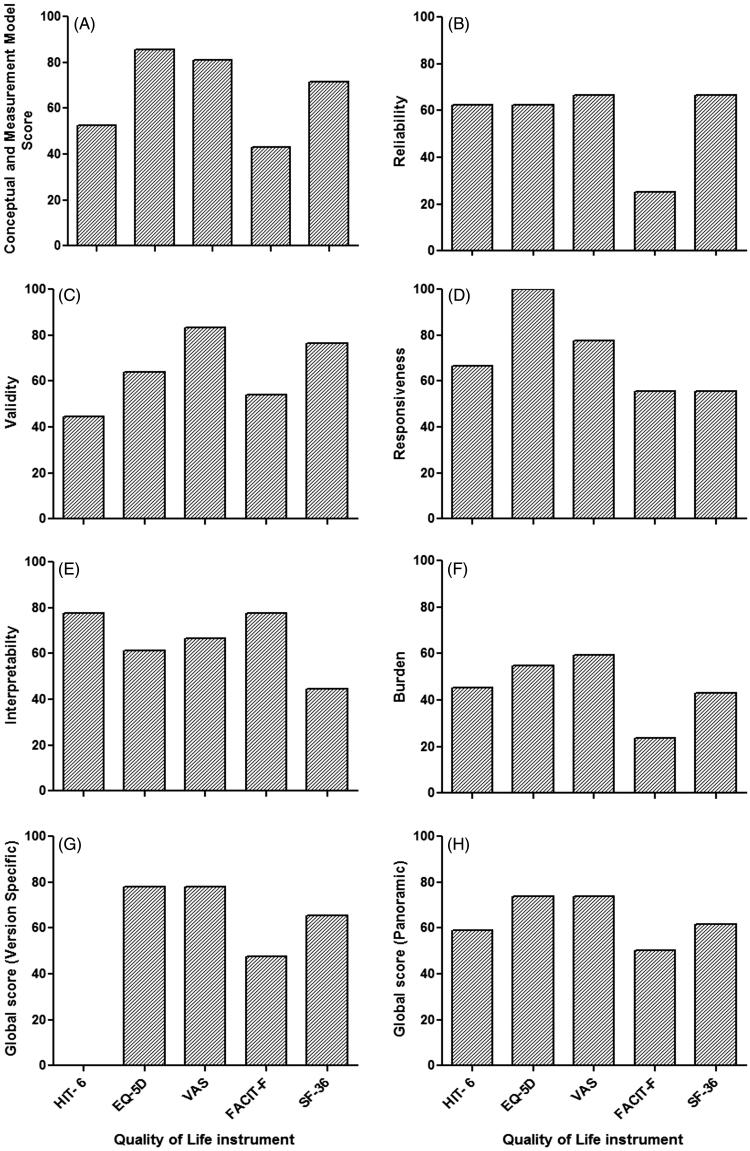
Overall ranking of instruments and their attribute-specific EMPRO scores. EMPRO scores ranged 0–100 (worst to best). EMPRO: Evaluating Measures of Patient-Reported Outcomes; EQ-5D: EuroQol five dimensions questionnaire; FACIT-F: Functional Assessment of Chronic Illness Therapy-Fatigue; SF-36: 36-item Short Form Health Survey; HIT- 6: Headache Impact Test-6; VAS: Visual Analogue Scale.

Detailed EMPRO results of the standardized evaluation are presented in Table 3 and summarized in Figure 4. The overall score, which summarizes the five attribute-specific scores were ranged from 73.83 (VAS) to 50.24 (FACIT-F). In the ‘conceptual and measurement model’ attribute, instruments scored from 85.71 (EQ-5 D) to 42.86 (FACT-F), with all instruments presenting scores higher than 50. ‘Reliability’ scores ranged from 66.67 (VAS and SF-36) to 25 (FACT-F) and 4/5 instruments scored above the threshold of 50. ‘Validity’ scores ranged from 83.33 (VAS) to 44.44 (HIT- 6), with only one instruments score below 50 (HIT- 6). In ‘Responsiveness’, instruments scored from 100 (EQ-5 D) to 55.56 (FACIT-F and SF-36) and all instruments scored more than 50. ‘Interpretability’ scores ranged from 77.78 (FACT-F and HIT-6) to 44.44 (SF-36) and only SF-36 instrument scored below the threshold of 50. ‘Burden’ scores were highest for VAS (59.52), followed by EQ-5 D (54.76), 45.24 (HIT-6) and 42.86 (SF-36) with FACIT-F presenting the lowest burden (23.81).

**Table 3.** Ratings of each EMPRO item and attribute for every quality of life instrument identified.

Attributes	HIT- 6^a^	EQ-5D^a^	VAS^a^	FACIT-F^a^	SF-36^a^
*Conceptual and measurement model*	52.38	85.71	80.95	42.86	71.43
Concept of measurement stated	++++	++++	++++	++++	++++
Obtaining and combining items described	++++	++++	++++	++	++++
Rationality for dimensionality and scales	+	++++	++++	+	++++
Involvement of target population	++++	++++	—	+++	—
Scale variability described and adequate	+	+++	++++	++	+++
Level of measurement described	+	++++	+++	+	+++
Procedures for deriving scores	++++	+++	++++	+++	++++
*Reliability*	62.50	62.50	66.67	25.00	66.67
Data collection methods described	++++	++++	++++	++	++++
Cronbach’s alpha adequate	+	+	++++	++	+++
Item Response Theory (IRT) estimates provided	+++	++++	—	—	—
Testing in different populations	++++	—	—	—	++++
Data collection methods described	+++	++++	+++	++	++++
Test–retest and time interval adequate	+++	+++	+++	++	+++
Reproducibility coefficients adequate	+	++	++	++	++
IRT estimates provided	+++	+++	+++	—	—
*Validity*	44.44	63.89	83.33	54.17	76.67
Content validity adequate	+	++	++++	+++	+
Construct/criterion validity adequate	++++	++++	++++	+++	++++
Sample composition described	++++	++	++++	++	++++
Prior hypothesis stated	+	++	+++	+++	+++
Rational for criterion validity	+	+++	N/A	N/A	N/A
Tested in different populations	++++	++++	N/A	N/A	++++
*Responsiveness*	66.67	100.00	77.78	55.56	55.56
Adequacy of methods	++++	++++	++++	+++	+++
Description of estimated magnitude of change	+++	++++	++++	+++	+++
Comparison of stable and unstable groups	++	++++	++	++	++
Interpretability	77.78	61.11	66.67	77.78	44.44
Rational of external criteria	+++	++++	++++	+++	+++
Description of interpretation strategies	++++	++	+++	++++	+++
How data should be reported stated	+++	++	++	++++	+
*Burden*	45.24	54.76	59.52	23.81	42.86
Skills and time needed	+++	+++	+++	++	+
Impact on respondents	+++	+++	++	++	++++
Not suitable circumstances	+++	++++	+	++	+
Resources required	+++	+++	+++	+++	++++
Time required	—	—	+++	—	—
Training and expertise needed	—	—	++++	—	—
Burden of score calculation	++	++++	++++	+	++++
*Alternative modes of administration*	—	75.00	—	16.67	41.67
Metric characteristics of alternative forms	—	+++	—	+	++
Comparability of alternative forms	—	+++	—	++	+++
*Cultural and language adaptations or translations*	—	100.00	100.00	27.78	83.33
Linguistic equivalence	—	++++	++++	++	++++
Conceptual equivalence	—	++++	++++	++	++++
Significant differences	—	++++	++++	+	+++
*Global score (Panoramic)*	59.09	73.81	73.83	50.24	61.70
*Global score (Version Specific)*	—	78.17	78.19	47.42	65.31

^a^** **++++: 4 (strongly agree);** **+++: 3;** **++: 2;** **+: 1 (strongly disagree), —: no information, N/A: Not Applicable. The higher the agreement the better the rating. EQ-5D: EuroQol five dimensions questionnaire; FACIT-F: Functional Assessment of Chronic Illness Therapy-Fatigue; SF-36: 36-item Short Form Health Survey; HIT-6: Headache Impact Test-6; VAS: Visual Analogue Scale.

PRO instrument viz. EQ-5 D, FACIT-F and SF-36 provide alternative modes of administration. Only EQ-5 D instrument scored the minimum threshold of EMPRO (75.00). In FACIT-F and SF-36 cases, the EMPRO scores were unable to reach the minimum threshold of 50 points (16.67 and 41.67, respectively) for ‘Alternative modes of administration’. However, no information was found for two instruments (HIT-6 and VAS) for ‘Alternative modes of administration’. EQ-5 D and VAS were well rated (100 points) for ‘Cultural and language adaptations or translations’, whereas SF-36 was rated as 83.33 for the same. FACIT-F instrument scored below the threshold of 50 for EMPRO (27.78) and there was no information available for HIT- 6 for ‘Cultural and language adaptations or translations’.

## 4. Discussion

Understanding and evaluation of the HRQoL in patients with aHUS plays an significant role in the development of new treatment regimen as well as health protocols. QoL is now becoming progressively perceived as an essential aspect for aHUS patients. Improving HRQoL should be considered while utilizing costly medications as a treatment option for prolonging life for a short length of time [47]. Eculizumab, a monoclonal antibody that specifically binds to C5 has been widely used worldwide for the treatment of aHUS [48]. The present study demonstrates that treatment with the eculizumab significantly improves HRQoL in patients with aHUS [27–29,41–46]. Moreover, it also provides symptomatic relief and improves the renal function as well as hematologic parameters of patients with aHUS [49].

To the best of our knowledge, only two reviews have reported the data of HRQoL in aHUS population [48,50]. However, none of the studies reported the performance of patient self-reported HRQoL instruments applicable for aHUS patients. In the present systematic review, we have reported the psychometric determinants properties of HRQoL instruments in patients with aHUS by assessing 10 studies which used five different PRO instruments. Furthermore, we have also provided the comparison amongst the PRO instruments based on EMPRO scoring via evaluating information related to the development process, metric properties and administrative issues.

Previously it has been established that the PRO instrument obtained the category of ‘recommended with provisos or alterations’ if it’s overall EMPRO score can achieve above the threshold of 50. Furthermore, if the EMPRO score is higher than 50.00 in every dimension, then instrument was categorized as ‘strongly recommended’. If the instrument is unable to achieve its overall EMPRO score above the threshold of 50, then it was categorized as ‘not recommend’. The PRO instrument obtained the category of ‘unsure’ if there was a discrepancy between reviewers [34].

Amongst the five instruments, most frequently used instrument was EQ-5 D and it had the best rating according to EMPRO standard criteria along with VAS. These generic HRQoL Instruments (EQ-5 D and VAS) have their overall scores higher than 50. Thus they are much satisfactory for assessing HRQoL in aHUS patients. Both instruments (EQ-5 D and VAS) scored higher than 50 in every dimension and achieved overall score higher than 70 (73.81 and 73.83, respectively). However, there was a couple of differences on the responsiveness and validity attributes. EQ-5 D also scored higher (100 points) in responsiveness than VAS, whereas in terms of validity VAS achieved higher score (83.33) than EQ-5 D. In congruence to the literature [34], our findings showed that EQ-5 D and VAS administration should be recommended as a generic instrument for assessing HRQoL in aHUS patients.

SF-36 obtained the third best rating in the overall summary score. It was at the top for ‘reliability’ along with VAS in our metric quality evaluation. Furthermore, it was also able to achieve above the threshold in EMPRO scoring for ‘conceptual and measurement model’ and ‘reliability’ but just passed the requirements of ‘responsiveness’. However, ‘interpretability’, ‘burden’ and ‘alternative modes of administration’ attributes unable to meet the threshold of EMPRO scoring. In the light of these results, our investigation showed that SF-36 could be ‘recommended with provisos or alterations’ in aHUS patients for determination of HRQoL.

TheSF-36 is a self-report questionnaire which determines eight multi-item variables which include physical functioning, social functioning, physical limitation of role, emotional limitation of role, pain, mental health, general health and vitality. The scale of 0–100 is used to record a score of each dimension. This scale is most popularly used to determine the functional status of the patients. Whereas, the Euroqol questionnaire is a quick response instrument which consists of five different elements, each with three levels [51]. Studies report that EQ-5 D provides more comparable results than SF-36 in the patients with various levels of perceived ill-health [52]. The finding of the present investigation also showed that EQ-5 D administration should be recommended than SF-36 for assessing HRQoL in aHUS patients and this is in line with the previous studies [52].

It has been well documented that EQ-5 D instrument is an extreme brevity and is been well characterized in large normal population studies [52]. When comparing the two instruments EQ-5 D and SF-36 based on health status, EQ-5 D provides insights about the current health status of patients, whereas SF-36 provides previous four weeks health status which might provide an idea of the relative health stability of patients' over a period. However, patients’ current health status gives a better idea about the disease state than a health state of several weeks ago. The pain and anxiety/depression largely affected the usual activities of the patient and these are greatly captured by EQ-5 D than SF-36. Additionally, SF-36 has a limited range of possible responses in the dimensions of physical or emotional factors. In the assessment of mental health problems, EQ-5 D has an advantage of capturing the response by simply asking the patient qualitatively about their anxious or depressed state.

The FACIT-F scale is a 13-item instrument designed to evaluate the impact of fatigue/tiredness daily activities and functioning in numerous chronic conditions [53,54]. It was originally designed to assess the fatigue in cancer patients where it showed good reliability and validity [54]. Further, it has been employed in an array of chronic diseases including systemic lupus erythematosus [55,56]; psoriatic arthritis [57]; chronic immune thrombocytopenia [58] and Parkinson’s disease [59]. HIT-6 is another disease-specific PRO instrument which was developed to measure pain. However, the low number of items in the questionnaire and inability to measure very mild headache impact may constituted a drawback for HIT-6 [60]. In our study, both instruments obtained a very high rating in ‘interpretability’, however, FACIT-F was unable to meet the minimal threshold of EMPRO scoring (except interpretability). Furthermore, HIT-6 and FACIT-F scored slightly above the overall threshold of acceptable results in our EMPRO minimum quality criterion. Thus, they were categorized as ‘not recommend’.

## 5. Limitations

Our present investigation possesses limitation as well and thus results of the present investigation should be interpreted by considering this limitation. Firstly, the available evidence from present investigation showed that HRQoL is significantly improved by eculizumab treatment in aHUS patients. However, this evidence was mainly based on prospective randomized studies and most of patients were 28 years (range 13–68 years). Further, real-world evidence studies with the more representative sample or long-term follow-up data are needed to validate this in daily clinical practice. Secondly, evaluation of PRO instrument on the basis of EMPRO is completely depended on quantity and quality of published literature. Furthermore, any unavailable or missing information in various attributes such as cultural adaptation, the burden of administration, validity, responsiveness or interpretation is considered as a worst possible rating in the EMPRO scoring algorithm may account for penalizes in the global EMPRO score. If more than half of the information is available, then overall EMPRO score should not determine to reduce this penalize of the global EMPRO score. Lastly, although the scoring of EMPRO was carried out by two independent reviewiers there may be a scope of bias by an individual expertise.

## 6. Conclusions

In conclusion, eculizumab significantly improves HRQoL in patients with aHUS. Evidence from present investigation support that EQ-5 D and VAS administration should be recommended as PRO instrument for assessing HRQoL in aHUS patients. Furthermore, EQ-5 D can be used in the evaluation of HRQoL aspects on an economic evaluation such as a cost-utility analysis and quality-adjusted life years (QALYs), where it would be necessary to map another instrument with it. However, their selection is entirely depending upon the objective and study requirements.

## 7. Implications for clinical practice

Our results suggest that EQ-5 D is the recommended PRO instrument for determination of HRQoL in aHUS patients treated with eculizumab. It would be implicated in case of economic evaluations for cost-utility analysis. However, determination of the quality of life in aHUS patient by using VAS and SF-36 can be achieved if the objective of study focuses attention on the items related to the validity attribute.

## Supplementary Material

Supplementary Figure

## References

[1] Gasser C, Gautier E, Siebenmann R. Hämolytisch-urämische Syndrome im Kindesalter[Hemolytic uremic syndromes in childhood Fifth Congress of the European Society of Hematology] In: Fünfter Kongress Der Europäischen Gesellschaft Für Hämatologie. Berlin, Heidelberg: Springer Science + Business Media; 1956 p. 787–788.

[2] Michael M, Elliott EJ, Craig JC, et al Interventions for hemolytic uremic syndrome and thrombotic thrombocytopenic purpura: a systematic review of randomized controlled trials. Am J Kidney Dis. 2009;53:259–272. 18950913 10.1053/j.ajkd.2008.07.038

[3] Taylor CM, Machin S, Wigmore SJ, et al Clinical practice guidelines for the management of atypical haemolytic uraemic syndrome in the United Kingdom. Br J Haematol. 2010;148:37–47. 19821824 10.1111/j.1365-2141.2009.07916.x

[4] Loirat C, Fremeaux-Bacchi V. Atypical hemolytic uremic syndrome. Orphanet J Rare Dis. 2011;6:60. 21902819 10.1186/1750-1172-6-60 PMC3198674

[5] Noris M, Remuzzi G. Atypical hemolytic-uremic syndrome. N Engl J Med. 2009;361:1676–1687. 19846853 10.1056/NEJMra0902814

[6] Kandhare AD, Patil MV, Bodhankar SLL. Arginine attenuates the ethylene glycol induced urolithiasis in ininephrectomized hypertensive rats: role of KIM-1, NGAL, and NOs. Ren Fail. 2015;37:709–721. 25682972 10.3109/0886022X.2015.1011967

[7] Fremeaux-Bacchi V, Fakhouri F, Garnier A, et al Genetics and outcome of atypical hemolytic uremic syndrome: a nationwide French series comparing children and adults. Clin J Am Soc Nephrol. 2013;8:554–562. 23307876 10.2215/CJN.04760512 PMC3613948

[8] Visnagri A, Kandhare AD, Bodhankar SL. Renoprotective effect of berberine via intonation on apoptosis and mitochondrial-dependent pathway in renal ischemia reperfusion-induced mutilation. Ren Fail. 2015;37:482–493. 25598236 10.3109/0886022X.2014.996843

[9] Kavanagh D, Richards A, Goodship T, et al Transplantation in atypical hemolytic uremic syndrome. Semin Thromb Hemost. 2010;36:653–659. 20865642 10.1055/s-0030-1262887

[10] Adil M, Kandhare AD, Ghosh P, et al Ameliorative effect of naringin in acetaminophen-induced hepatic and renal toxicity in laboratory rats: role of FXR and KIM-1. Ren Fail. 2016;38:1007–1020. 27050864 10.3109/0886022X.2016.1163998

[11] Zuber J, Fakhouri F, Roumenina LT, et al Use of eculizumab for atypical haemolytic uraemic syndrome and C3 glomerulopathies. Nat Rev Nephrol. 2012;8:643–657. 23026949 10.1038/nrneph.2012.214

[12] Saland JM, Ruggenenti P, Remuzzi G, et al Liver-kidney transplantation to cure atypical hemolytic uremic syndrome. J Am Soc Nephrol. 2009;20:940–949. 19092117 10.1681/ASN.2008080906

[13] US Food and Drug Administration Soliris® (eculizumab) [prescribing information]. Cheshire (CT): Alexion Pharmaceuticals, Inc; 2014.

[14] European Medicines Agency Soliris® (eculizumab) [prescribing information]. Paris, France: Alexion Europe; 2015.

[15] Ghosh P, Kandhare AD, Kumar VS, et al Determination of clinical outcome and pharmacoeconomics of anti–rheumatoid arthritis therapy using CDAI, EQ–5D–3L and EQ–VAS as indices of disease amelioration. Asian Pac J Trop Dis. 2012;2:S671–S678.

[16] Shivakumar V, Kandhare AD, Rajmane AR, et al Estimation of the long-term cardiovascular events using ukpds risk engine in metabolic syndrome patients. Indian J Pharm Sci. 2014;76:174–178. 24843193 PMC4023289

[17] Ghosh P, Kandhare AD, Raygude KS, et al Determination of the long term diabetes related complications and cardiovascular events using UKPDS risk engine and UKPDS outcomes model in a representative western Indian population. Asian Pac J Trop Dis. 2012;2:S642–S650.

[18] Centers for Disease Control Prevention Measuring healthy days: population assessment of health-related quality of life. Atlanta: CDC; 2000.

[19] Dole S, Kandhare AD, Ghosh P, et al Homeopathic analgesic formulations: a critical appraisal of evidence. J Pharm Biomed Sci. 2012;22:1–6.

[20] Ghosh P, Kandhare AD, Raygude KS, et al Cigarette smoking and H. pylori infection: a meta-analysis of literature. Pharm Lett. 2012;4:128–134.

[21] Ware JE, Kosinski M, Bjorner JB, et al User’s manual for the SF-36v2 Health Survey. 2nd ed. Lincoln (RI): Quality Metric; 2008.

[22] Wenger NK, Mattson ME, Furberg CD, et al Assessment of quality of life in clinical trials of cardiovascular therapies. Am J Cardiol. 1984;54:908–913. 6333175 10.1016/s0002-9149(84)80232-5

[23] Gosavi TP, Kumar VS, Kandhare AD, et al A comprehensive metaanalysis and systematic review on effect of genistein on metabolic syndrome. Pharmacologia. 2015;5:120–126.

[24] Gosavi T, Ghosh P, Kandhare A, et al Unwrapping homeopathic principles in the wake of research: serendipity, placebo or true therapeutic milestones. Pharmacologyonline. 2011;1:894–906.

[25] Food and Drug Administration Guidance for industry: patient-reported outcome measures: use in medical product development to support labeling claims. Fed Reg. 2009;74:65132–65133.

[26] Gosavi T, Kandhare A, Raygude K, et al Evaluation of clinical outcome in arthritis with AIMS2. Deccan J Pharmacol. 2011;2:10–30.

[27] Legendre CM, Licht C, Muus P, et al Terminal complement inhibitor eculizumab in atypical hemolytic-uremic syndrome. N Engl J Med. 2013;368:2169–2181. 23738544 10.1056/NEJMoa1208981

[28] Greenbaum LA, Fila M, Ardissino G, et al Eculizumab is a safe and effective treatment in pediatric patients with atypical hemolytic uremic syndrome. Kidney Int. 2016;89:701–711. 26880462 10.1016/j.kint.2015.11.026

[29] Licht C, Greenbaum LA, Muus P, et al Efficacy and safety of eculizumab in atypical hemolytic uremic syndrome from 2-year extensions of phase 2 studies. Kidney Int. 2015;87:1061–1073. 25651368 10.1038/ki.2014.423 PMC4424817

[30] Mokkink LB, Terwee CB, Patrick DL, et al The COSMIN checklist for assessing the methodological quality of studies on measurement properties of health status measurement instruments: an international Delphi study. Qual Life Res. 2010;19:539–549. 20169472 10.1007/s11136-010-9606-8 PMC2852520

[31] Valderas JM, Ferrer M, Mendivil J, et al Development of EMPRO: a tool for the standardized assessment of patient-reported outcome measures. Value Health. 2008;11:700–708. 18194398 10.1111/j.1524-4733.2007.00309.x

[32] Garin O, Herdman M, Vilagut G, et al Assessing health-related quality of life in patients with heart failure: a systematic, standardized comparison of available measures. Heart Fail Rev. 2014;19:359–367. 23681849 10.1007/s10741-013-9394-7

[33] Schmidt S, Ferrer M, Gonzalez M, et al Evaluation of shoulder-specific patient-reported outcome measures: a systematic and standardized comparison of available evidence. J Shoulder Elbow Surg. 2014;23:434–444. 24406123 10.1016/j.jse.2013.09.029

[34] Maratia S, Cedillo S, Rejas J. Assessing health-related quality of life in patients with breast cancer: a systematic and standardized comparison of available instruments using the EMPRO tool. Qual Life Res. 2016;25:2467–2480. 27048496 10.1007/s11136-016-1284-8

[35] Schmidt S, Garin O, Pardo Y, et al Assessing quality of life in patients with prostate cancer: a systematic and standardized comparison of available instruments. Qual Life Res. 2014;23:2169–2181. 24748557 10.1007/s11136-014-0678-8 PMC4155169

[36] Liberati A, Altman DG, Tetzlaff J, et al The PRISMA statement for reporting systematic reviews and meta-analyses of studies that evaluate healthcare interventions: explanation and elaboration. BMJ. 2009;339:b2700. 19622552 10.1136/bmj.b2700 PMC2714672

[37] Kandhare AD, Mukherjee A, Ghosh P, et al Efficacy of antioxidant in idiopathic pulmonary fibrosis: a systematic review and meta-analysis. Excli J. 2016;15:636–651. 28096793 10.17179/excli2016-619 PMC5225735

[38] Cochrane Cochrane handbook for systematic reviews of interventions 5.0. 0. Indianapolis (IN): Cochrane Collaboration; 2008.

[39] van der Have M, van der Aalst KS, Kaptein AA, et al Determinants of health-related quality of life in Crohn’s disease: a systematic review and meta-analysis. J Crohns Colitis. 2014;8:93–106. 23746864 10.1016/j.crohns.2013.04.007

[40] Saultz JN, Wu HM, Cataland S. Headache prevalence following recovery from TTP and aHUS. Ann Hematol. 2015;94:1473–1476. 26063190 10.1007/s00277-015-2411-2

[41] Muus P, Licht C, Goodship TH, et al Eculizumab (ECU) significantly improves health-related quality of life (hrqol) in patients with atypical hemolytic uremic syndrome (aHUS). Blood. 2011;118:4772–4772.

[42] Licht C, Muus P, Legendre CM, et al Eculizumab (ECU) safety and efficacy in atypical hemolytic uremic syndrome (aHUS) patients with long disease duration and chronic kidney disease (CKD): 2-year results. Blood. 2012;120:985–985.22692510

[43] Greenbaum LA, Fila M, Ardissino G, et al Eculizumab inhibits thrombotic microangiopathy and improves renal function in pediatric patients with atypical hemolytic uremic syndrome: 1-year update. Blood. 2014;124:4986–4986.

[44] Greenbaum L, Legendre CM, Babu S, et al Eculizumab (ECU) in atypical hemolytic uremic syndrome (aHUS) patients with progressing thrombotic microangiopathy (TMA): 2-year data. Blood. 2012;120:2084–2084.

[45] Loirat C, Babu S, Furman R, et al Eculizumab efficacy and safety in patients with atypical hemolytic uremic syndrome (aHus) resistant to plasma exchange/infusion. Poster session presented at: 16th Congress of European Hematology Association (EHA), London, United Kingdom; 2011.

[46] Fakhouri F, Hourmant M, Campistol JM, et al Terminal complement inhibitor eculizumab in adult patients with atypical hemolytic uremic syndrome: a single-arm, open-label trial. Am J Kidney Dis. 2016;68:84–93. 27012908 10.1053/j.ajkd.2015.12.034

[47] Gunda P, Kandhare A, Nikoglou E, et al A cost per responder analysis of secukinumab vs adalimumab based on a matching-adjusted indirect comparison for the treatment of ankylosing spondylitis from a German payer perspective. Value Health. 2016;19:A537.

[48] Palma LM, Langman CB. Critical appraisal of eculizumab for atypical hemolytic uremic syndrome. J Blood Med. 2016;7:39–72. 27110144 10.2147/JBM.S36249 PMC4835139

[49] Riedl M, Hofer J, Giner T, et al Monitoring eculizumab treatment in patients with atypical hemolytic uremic syndrome (aHUS) by measearing TCC capacity and C3a concentration. Paper presented at: Pediatric Nephrology, Shanghai, China; 2013.

[50] Rathbone J, Kaltenthaler E, Richards A, et al A systematic review of eculizumab for atypical haemolytic uraemic syndrome (aHUS). BMJ Open. 2013;3:e003573. 10.1136/bmjopen-2013-003573 PMC3822313 24189082

[51] Myers C, Wilks D. Comparison of EuroQol EQ-5D and SF-36 in patients with chronic fatigue syndrome. Qual Life Res. 1999;8:9–16. 10457734 10.1023/a:1026459027453

[52] Brazier J, Jones N, Kind P. Testing the validity of the EuroQol and comparing it with the SF-36 health survey questionnaire. Qual Life Res. 1993;2:169–180. 8401453 10.1007/BF00435221

[53] Cella D, Lai JS, Chang CH, et al Fatigue in cancer patients compared with fatigue in the general United States population. Cancer. 2002;94:528–538. 11900238 10.1002/cncr.10245

[54] Yellen SB, Cella DF, Webster K, et al Measuring fatigue and other anemia-related symptoms with the Functional Assessment of Cancer Therapy (FACT) measurement system. J Pain Symptom Manage. 1997;13:63–74. 9095563 10.1016/s0885-3924(96)00274-6

[55] Kosinski M, Gajria K, Fernandes AW, et al Qualitative validation of the FACIT-fatigue scale in systemic lupus erythematosus. Lupus. 2013;22:422–430. 23423250 10.1177/0961203313476360

[56] Lai JS, Beaumont JL, Ogale S, et al Validation of the functional assessment of chronic illness therapy-fatigue scale in patients with moderately to severely active systemic lupus erythematosus, participating in a clinical trial. J Rheumatol. 2011;38:672–679. 21239746 10.3899/jrheum.100799

[57] Chandran V, Bhella S, Schentag C, et al Functional assessment of chronic illness therapy-fatigue scale is valid in patients with psoriatic arthritis. Ann Rheum Dis. 2007;66:936–939. 17324972 10.1136/ard.2006.065763 PMC1955111

[58] Signorovitch J, Brainsky A, Grotzinger KM. Validation of the FACIT-fatigue subscale, selected items from FACT-thrombocytopenia, and the SF-36v2 in patients with chronic immune thrombocytopenia. Qual Life Res. 2011;20:1737–1744. 21533818 10.1007/s11136-011-9912-9

[59] Hagell P, Hoglund A, Reimer J, et al Measuring fatigue in Parkinson's disease: a psychometric study of two brief generic fatigue questionnaires. J Pain Symptom Manage. 2006;32:420–432. 17085268 10.1016/j.jpainsymman.2006.05.021

[60] Nachit‐Ouinekh F, Dartigues JF, et al Use of the headache impact test (HIT‐6) in general practice: relationship with quality of life and severity. Eur J Neurol. 2005;12:189–193. 15693807 10.1111/j.1468-1331.2004.00934.x

