# Supplementary figures and images for: Evaluation of health-related quality of life in hemolytic uraemic syndrome patients treated with eculizumab: a systematic evaluation on basis of EMPRO
Source: Ren Fail. 2018 Jan 24;40(1):107–18. doi: 10.1080/0886022X.2018.1427110 (PMC6014301; doi:10.1080/0886022X.2018.1427110)

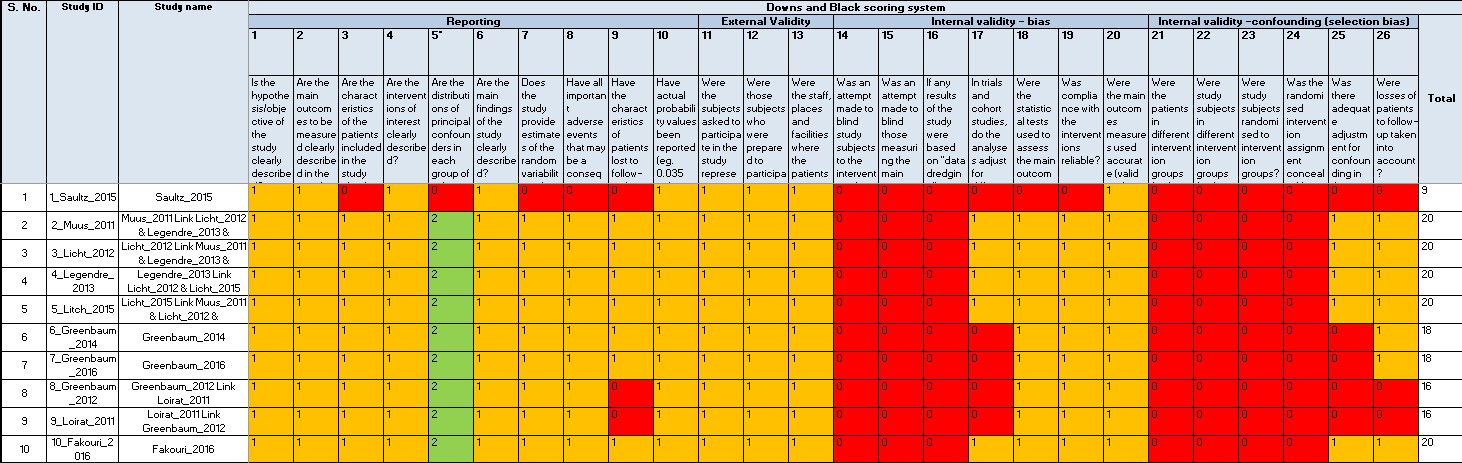

Supplement: Supplementary Figure [file IRNF_A_1427110_SM8785.jpg]
